# Length of Stay in Patients With Central Line-Associated Bloodstream Infection at a Tertiary Hospital in the Kingdom of Saudi Arabia

**DOI:** 10.7759/cureus.10820

**Published:** 2020-10-06

**Authors:** Naif H Alotaibi, Abdulrahman Barri, Muhammad A Elahi

**Affiliations:** 1 Infectious Disease, King Saud University, College of Medicine, Riyadh, SAU; 2 Internal Medicine, King Saud University, College of Medicine, Riyadh, SAU; 3 Internal Medicine, Alfaisal University College of Medicine, Riyadh, SAU

**Keywords:** clabsi, central line associated bloodstream infection, los, length of stay

## Abstract

Objective

To examine the impact of central line-associated bloodstream infection (CLABSI) on hospital length of stay (LOS) and to identify the factors associated with prolonged LOS.

Methods

The research setting was King Saud University Medical City (KSUMC) in Riyadh, Kingdom of Saudi Arabia. A retrospective cohort design was applied with a sample of adult CLABSI patients. Patients developed CLABSI following central line insertion at KSUMC between March 2016 and February 2018.

Results

The CLABSI-related prolongation of LOS was 13.13 ± 9.53 days for a total of 283 patients. This figure rose for patients with any CLABSI-related sequela, and the result was statistically significant (p<0.033). It was also significantly higher in patients with delayed central line removal (p<0.001). A patient’s setting (i.e., in the intensive care unit prior to or following infection) was not a factor associated with prolonged LOS. Nevertheless, the requirement for inotropes after the infection was linked to prolonged LOS in a statistically significant way (p<0.048).

Conclusions

For ill patients who need hemodynamic support following infection, CLABSI can significantly increase hospital LOS. Delayed decisions or slow central line removal are associated with significant increases in LOS.

## Introduction

Central venous catheters (CVCs) are often essential when caring for critically ill patients, as they allow for the safe administration of intravenous (IV) medications that cannot be administered peripherally. However, CVCs are potential portals for localized and systemic bloodstream infections.

Healthcare-associated infections contribute to high morbidity, mortality, and healthcare costs. The rising use of central lines in routine clinical practice has made central line-associated bloodstream infections (CLABSI) a leading cause of preventable healthcare infections [1]. A 2017 report from six hospitals in the Kingdom of Saudi Arabia (KSA), Oman, and Bahrain found that the overall CLABSI rate was 3.1/1,000 central line days [2]. Moreover, a KSA-based study involving 12 Ministry of Health hospitals reported that CLABSI rates varied from 2.2 to 10.5/1,000 central line days between 2013 and 2016 [3].

CLABSI is defined by the Centers of Disease Control and Prevention (CDC) surveillance criteria that identify bloodstream infections in patients with CVCs who have no obvious secondary source of bacteremia [4]. Laboratory-confirmed bloodstream infection is reported as CLABSI when the patient has a central line inserted for more than two days, and when the central line is in place on the day of (or on the day prior to) the first sign or symptom that meets the definition [5]. The infection should not be attributable to any other infection the patient may have [6].

CLABSI has a substantial impact on patient morbidity and mortality [7], as well as on healthcare systems, where it generates an economic burden prolonging hospital length of stay (LOS) [8]. Prolonged LOS typically leads to greater fixed costs in terms of beds, facilities, staffing, and equipment. These costs cannot be easily eliminated, and although quantifiable cash savings are generally not obtained by avoiding CLABSI in hospitals, preventing such infections allows hospital resources to be allocated to more productive areas [9]. Hence, excess LOS must be estimated based on the number of bed days and resources that would have been conserved using preventive measures [10].

CLABSI prevention relies on multiple strategies, considering both insertion-related and catheter-related factors. Insertion-related factors include insertion under full sterile technique, and catheter-related factors include the use of antibiotic-impregnated catheters and suture-less securement devices, along with effective infectious control programs [11].

## Materials and methods

Research setting

This retrospective cohort study was undertaken at a tertiary hospital, King Saud University Medical City (KSUMC), in Riyadh in the Kingdom of Saudi Arabia (KSA). 

Participants

The study’s target population consisted of adult patients ≥ 18 years old who received a central line insertion at KSUMC and who developed CLABSI between March 2016 and February 2018.

Inclusion criteria

A list of all positive cultures from central lines was obtained from the microbiology lab records. The list was then filtered to include only patients who had an eligible bloodstream infection (BSI) organism and an eligible central line. Eligible lines had to have been in place for at least 48 hours prior to culture or 24 hours after central line removal. 

Data collection

After receiving training on the diagnostic criteria for the Centers for Disease Control and Prevention (CDC) CLABSI, internal medicine physicians at KSUMC obtained data on demographics, central line characteristics, admission characteristics, treatment, and the clinical outcomes of episodes from electronic medical records. Length of time was defined either as the period required to treat CLABSI or its sequelae from the time of establishing the diagnosis or the period required for new central line insertion if the insertion led to delayed hospital discharge.

Statistical analysis

Statistical analysis was performed using Statistical Package for the Social Sciences (SPSS) version 22.0 (IBM Corp, Armonk, NY). Continuous data were expressed in the form mean ± standard deviation (SD), and discrete data were expressed as counts and percentages. The probability of increased LOS was determined using the student's t-test for independent groups, analysis of variance (ANOVA), and the Pearson correlation coefficient, as appropriate. P-values of less than or equal to 0.05 were considered significant.

## Results

A total of 283 patients (52.8 ± 20.5 years) were involved in the study, over half of whom were male (n = 157, 55.5%). A total of 138 patients (48.8%) were admitted to the intensive care unit (ICU) either before developing CLABSI or due to their infection (Table 1). CLABSI prevention bundles at the time of insertion were only applied in three patients (1.1%) and were used as maintenance in only 89 (31.4%) (Table 2). Most patients had permanent central lines (n = 179, 63.3%), and for 13 patients (6.57%), central lines were changed using a guidewire after developing CLABSI (Table 2). Table 3 shows the distribution of indications of central line positions.

**Table 1:** Admission characteristics compared to length of stay SD: standard deviation

Variables	Value	Length of stay (Mean±SD)	P-value
Age	Correlation	-0.076	0.21
Gender	Male	12.78±9.29	0.478
	Female	13.60±9.29	
ICU admission	Yes	12.67±9.03	0.212
	No	16.21±32.00	
SD: standard deviation			

**Table 2:** Central line characteristics

Variables	Value	N (%)	
			Bundle insertion	Yes	3 (1.1%)	
No	280 (98.9%)		
Bundle maintenance	Yes	89 (31.4%)	
	No	194 (68.6%)	
Number of central lines	Single	247 (87.3%)	
	Multiple	36 (12.7%)	
Type of central line	Permanent	179 (63.3%)	
	Temporary	104 (36.7%)	
Usage of a guidewire to replace the line	Yes	13 (6.57%)	
	No	185 (93.43%)	

**Table 3:** Indications of central line insertion TPN: total parenteral nutrition

Dialysis	116 (41.1%)
Inotrope	53 (18.8%)
Placement of venous access line, no other peripheral sites	50 (17.7%)
Alternative to repetitive venous cannulations	27 (9.6%)
Chemotherapy	25 (8.9%)
Plasmapheresis	2 (0.7%)
Irritant and vesicant medications	34 (12.1%)
TPN	17 (6%)
Blood transfusion	3 (1.1%)
Other than peripheral line	2 (0.7%)

A total of 208 patients (75.4%) were discharged without any complications. However, 52 (18.8%) died within 30 days of the infection and 16 (5.8%) developed a CLABSI-related complication. The presence of CLABSI-related sequela was statistically associated with increased LOS (p<0.033) (Table 4). Regarding the patients’ mean times in terms of developing CLABSI after admission, after central line insertion, and at the time of removal of central lines after infection and LOS were 62.28 ± 246.36 days, 66.54 ± 161.43 days, 3.61 ± 4.95 days, and 13.13 ± 9.53 days, respectively (Table 5).

**Table 4:** End point of patients compared to length of stay

Variables	Value	N (%)	P-value
End point	Discharge	208 (75.4%)	-
	Sequela	16 (5.8%)	0.033
	Deaths	52 (18.8)	-

**Table 5:** Central line characteristics compared to length of stay CLABSI: central line-associated bloodstream infection

Variables	Length of stay (Correlation)	P-value
Mean #days to have CLABSI after line insertion	0.114	0.06
Mean #days to have CLABSI after admission	0.046	0.446
Mean #days to death after developing CLABSI	0.927	0.000
Mean #days to have line removal after CLABSI	0.23	0.001

No statistically significant differences were identified between LOS and gender (p<0.478). Moreover, LOS, in turn, was not significantly affected by ICU admission (p<0.212). Additionally, no statistically significant correlation was appreciable between LOS and the age of patients (p<0.21) (Table 1). Interestingly, LOS was significantly higher among patients who required inotrope treatment after culture (p<0.048) (Table 6). A significantly positive correlation was observed between LOS and the time of central line removal (p<0.001) after the development of CLABSI. Furthermore, a significant and positive correlation was observed between LOS and the time of central line removal (p<0.001) after developing CLABSI, as well as the time of death (p<0.0001), with a lower LOS as compared to patients who finished a full course of antibiotics (Table 5).

**Table 6:** Inotrope and intubation requirements compared to length of stay SD: standard deviation, CLABSI: central line-associated bloodstream infection

Variables	Value	Length of stay (Mean±SD)	P-value	
				Inotrope before CLABSI	Yes	11.88±11.23	0.437	
No	13.35±9.23						
Inotrope after CLABSI	Yes	11.60±10.14	0.048	
	No	13.97±9.11		
Inotrope requirement	Increased	10.40±11.77	0.395	
	Decreased	12.00±6.81		
	Same	19.75±8.84		
	Not required before culture	11.63±9.49		
Intubation before CLABSI	Yes	12.51±9.29	0.437	
	No	13.45±9.67		
Intubation after CLABSI	Yes	12.18±9.42	0.163	
	No	13.81±9.59		
Intubation requirement	Increased	11.92±11.25	0.921	
	Decreased	14.17±7.27		
	Same	12.58±8.21		
	Not required before culture	11.50±9.75		

## Discussion

In studies addressing the cost-effectiveness of CLABSI-preventing interventions, hospital LOS is a cardinal outcome [10]. CLABSI-associated increases in LOS contribute significantly to higher hospital costs [12]. In turn, this exhausts hospital resources, worsens the patient experience, and places the patient at further risk of subsequent hospital-acquired infections [13].

This study found that CLABSI-associated LOS amounted to an average of 13.13 ± 9.53 days. This number was significantly related to the total number of deaths (p<0.0001), having lower LOS than patients who completed a full course of antibiotics. This is comparable to the LOS observed in the multicenter study undertaken by Rosenthal et al., which involved 69 International Nosocomial Infection Control Consortium (INICC) tertiary care hospital ICUs in 11 countries [14]. The researchers found that LOS in CLABSI patients was 14.5 ± 3.08 days, representing 9.8 days in excess LOS. Notably, ICU patients were not at risk of higher LOS as compared to patients on general wards.

In Jia et al.’s recent study, which was undertaken across 68 hospitals, the results suggested that LOS may be influenced by an organism’s sensitivity to antibiotics. For non-drug-resistant organism infections, the LOS was 12.2 days, whereas it was 14 days for multi-drug-resistant organism infections in hospital-acquired bloodstream infections [15].

It is notable that, in this study, the presence of CLABSI-related complications was associated with a significant increase in LOS. Another key finding was that the time taken to remove the infected central line was significantly associated with increased LOS (p<0.001). This time was sometimes affected by the physician’s decision regarding the necessity for central line access and, at other times, by the slow process of removing the line in the appropriate place.

This study also indicated that age and gender were not significantly related to CLABSI-associated LOS. These findings are consistent with those of Atilla et al. in that CLABSI was not associated with age and gender [16]. This finding aids in removing any potential gender or age bias during CLABSI surveillance. Inotrope administration after culture was significantly associated with increased LOS.

In the study undertaken by Cardenas-Garcia et al., inotropes administration (e.g., norepinephrine and dopamine) via peripheral intravenous access was identified as both feasible and safe due to the lower risk of extravasation as compared to central venous access [17]. This suggests that the administration of vasoactive medications should not lead to an automatic insertion of central venous lines.

CLABSI has been characterized accurately as a partially preventable, device-associated, and hospital-acquired infection. Many strategies may be effective in reducing the incidence of CLABSI infection, including the passive strategy, which involves quick feedback through following surveillance protocols [18]. Dedicated infection control programs and effective surveillance, in comparison to regimes lacking such programs, were found to reduce the rate of infection to 32% [19]. Hence, the Society for Healthcare Epidemiology in America considers surveillance practices as a category 1 recommendation [20]. Recently, the bundle concept was introduced, focusing on reducing the rate of CLABSI by applying preventive measures at the time of central line insertion, and as surveillance during the hospital stay. In this study, CLABSI prevention surveillance bundles were only used in one-third of the patients, and they were seldom used at the time of insertion. Unfortunately, this study had a few limitations. Firstly, there were some important variables that could not be investigated and controlled. These included the severity of illness of patients and its possible effect on the rate of mortality as well as staff characteristics and qualifications. Secondly, this study did not compare the LOS of patients admitted with the same disease and developed CLABSI to those who did not.

## Conclusions

LOS is significantly increased in hemodynamically unstable patients with CLABSI. Once identified, prompt central line removal is needed to prevent increased LOS.
